# Bacterial species isolated from cats with lower urinary tract infection and their susceptibilities to cefovecin

**DOI:** 10.1186/s13620-015-0030-9

**Published:** 2015-02-12

**Authors:** Banu Dokuzeylül, Beren Başaran Kahraman, Alper Bayrakal, Belgi Diren Siğirci, Baran Çelik, Serkan Ikiz, Abdullah Kayar, M Erman OR

**Affiliations:** Department of Internal Medicine, Faculty of Veterinary Medicine, Istanbul University, 34320 Avcılar, Istanbul, Turkey; Department of Microbiology, Faculty of Veterinary Medicine, Istanbul University, 34320 Avcılar, Istanbul, Turkey

**Keywords:** Cat, Urinary tract infection, Urine culture, Antimicrobial susceptibility, Cefovecin

## Abstract

**Background:**

The aim of this study was to determine the bacterial species recovered from 61 cats with lower urinary tract infection (LUTI), and their susceptibility to cefovecin *in vitro*.

**Results:**

The clinical signs and final clinical diagnosis for cats with confirmed LUTI were also reported. After physical examination of the cats, urine samples including ≥5-6 leucocytes in microscopic evaluation were cultured using bacteriological techniques. The isolates were identified by conventional microbiological methods and tested for in vitro susceptibility using the Kirby-Bauer disc diffusion method recommended by the Clinical Laboratory Standards Institute. Bacterial growth was observed in 16 of 61 urine samples. Antimicrobial susceptibility tests showed that 13 of 16 (81%) isolates were susceptible to cefovecin. The most frequently isolated bacterium from cats with signs of lower urinary tract infection, was *Escherichia coli*.

**Conclusion:**

Cefovecin was found to be effective in cats with LUTI. Because cefovecin is a new antimicrobial agent in veterinary medicine, there are only few studies about urine culture of cats with LUTI. It is the first study on *in vitro* activity of cefovecin against bacterial isolates from cats with lower urinary infections in Istanbul, Turkey.

## Background

Lower urinary tract infections (LUTI) are rarely seen in cats, dogs and human beings. Various lower urinary tract disorders can predispose to opportunistic infections as a complication of the underlying disease or its treatment, while bacteria can be the initial cause [1].

Urine culture is the gold standard used to confirm the diagnosis of urinary tract infection (UTI). The urine sample used for this purpose should be obtained by cystocentesis to avoid bacterial contamination from the lower urogenital tract flora [2].

In urinary tract infections of cats and dogs, the most commonly isolated bacterial species were reported as *Escherichia coli, Proteus* spp., *Staphylococcus* spp. and *Streptococcus* spp., although the prevalence of the various species varied considerably [3,4].

Cephalosporins belong to the beta-lactam group of antibiotics and they are originally derived by hydrolysis from the natural compound of Cephalosporin C. This class is bactericidal and acts by inhibiting the synthesis of the peptidoglycan layer of the bacterial cell wall through binding to the penicillin binding protein (PBP) [5].

Cefovecin sodium [Convenia®; Pfizer Animal Health; USA] is a newly developed, semi-synthetic, extended-spectrum injectable third-generation cephalosporin administered at 8 mg/kg subcutaneously (SC) for the treatment of UTI and skin and soft tissue infections in dogs and cats and it has been approved for subcutaneous (SC) injections in cats since 2006 in EU and 2008 in USA [4,6-9].

Third generation cephalosporins are generally less active than members of the first or second generation formulations against gram-positive organisms (e.g., *Streptococcus* spp. or *Staphylococcus* spp.) [5]. Stegemann *et al.* [4] have reported that cefovecin showed good activity against Gram-negative organisms isolated from dogs and cats, including *Escherichia coli, Pasteurella multocida, Klebsiella* spp. (including *K. pneumonia*), *Enterobacter* spp. and anaerobic-growing pathogens *Fusobacterium* spp.*, Bacteriodes* spp., *Prevotella oralis*. However it was not effective against most *Pseudomonas aeruginosa* isolates.

The aim of this study was to determine the bacterial species recovered from cats with LUTI, and their susceptibility to cefovecin *in vitro*. The clinical signs and final clinical diagnosis for cats with confirmed LUTI were also reported.

## Methods

### Samples

In this study, 90 cats with one or more urinary clinical signs such as stranguria, haematuria, pollakiuria, inappropriate urination, excessive licking of the genital area and frequent and/or prolonged attempts to urinate were physically examined at the Department of Internal Medicine, Faculty of Veterinary Medicine, Istanbul University and their anamnesis was gathered. Complete Blood Count (CBC), blood serum biochemistry (Serum glucose, blood urea nitrogen (BUN), creatinine, alanine aminotransferase (ALT), aspartate aminotransferase (AST)) and urine analyses were performed in all patients. Twenty-nine cats were excluded from the study because antimicrobial treatment had already commenced in private veterinary clinics prior to our physical examination. Sixty-one cats with no antimicrobial treatment and including ≥5-6 leucocytes in urine microscopic examination were included in the study. The examination focussed on the presence of pyuria (≥5 white blood cells/high magnification (40x objective; high-power field, (hpf)) which were indicator of LUTI. The cats in the sample group were from different breeds: mixed (n:38), Persian (n:11), Siamese (n:5), Turkish Van (n:3), Turkish Angora (n:4). Forty-one cats were male, 20 cats were female. Five of the cats were one year old or younger, 38 between 2–7 years old and 18 were 8 years old or older. Samples of 5 ml of urine were collected by ultrasound-guided cystocentesis. Cats were restrained in lateral recumbency, the caudal abdomen area was cleaned with alcohol then the needle was inserted. Urine samples for culture and antimicrobial susceptibility tests were sent to the laboratory within 1 hour, stored in cooling boxes.

### Medical imaging

Abdominal radiography and ultrasonography were also performed to diagnose underlying urinary diseases/disorders of the cats. Abdominal ultrasonography was performed using a 3.75-MHz convex transducer (Schimadzu 350-A, Shimadzu Corporation, Kyoto, Japan).

### Culture

The samples were sent for bacteriological examination to the Laboratory of the Microbiology Department of Istanbul University, Faculty of Veterinary Medicine. Urine samples were inoculated onto nutrient agar supplemented with 7% sheep blood (blood agar) and MacConkey agar plates. While the MacConkey agar plates were incubated aerobically, the blood agar plates were incubated under aerobic and microaerobic conditions at 37°C for 7 days. The colonies were examined macroscopically and then microscopically using Gram staining. Biochemical identification was performed by conventional methods and all the isolates were confirmed with API systems (BioMérieux, SA, Marcy I’Etolie, France) [10,11]. A bacterial count of more than 10^3^ cfu/ml was considered diagnostic of UTI [9]. Cultures with no growth after 7 days were interpreted as negative.

### Antimicrobial susceptibility tests

The antibiotic susceptibility tests were performed according to the Kirby-Bauer method recommended by the Clinical Laboratory Standards Institute (CLSI) to select the optimal antimicrobial agent for treatment [12]. The zone of inhibition around the disk (30 μg cefovecin) was measured. The inhibition zone of ≥ 23 mm was considered as susceptible, while 20–22 mm as intermediate and ≤ 19 mm as resistant [6,12].

### Statistical analyses

The results were analysed with the SPSS 13.0 programme. The Chi-squared test was used for the comparisons of gender groups and age groups with respect to bacterial growth. Differences were considered significant at *p* < 0.05.

## Results

### Clinical signs

The most common presenting clinical signs of bacterial lower urinary tract infection in cats were pollakiuria (n = 41) followed by stranguria and haematuria, respectively.

### Clinical disorders associated with lower urinary tract signs

Disorders and the number of cats involved are given in Table 1. Sixteen culture-positive cats were diagnosed with the following conditions: urethral plaque (n:4), feline idiopathic cystitis (n:1), haemorrhagic cystitis (n:2), bladder stones (n:1), acute renal failure (n:3), chronic renal failure (n:2), diabetes mellitus (n:1), other diseases (n:2). The final diagnosis was reached following anamnesis, physical examination, blood and urine analysis, medical imaging and urine culture.

**Table 1:** **Diseases/disorders determined in 61 cats with lower urinary tract signs**

**Disorders**	**Number of cats**	**Culture-positive cats**
**Haemorrhagic cystitis**	8	2
**Polypoid cystitis**	3	-
**Feline idiopathic cystitis**	4	1
**Transitional cell carcinoma (TCC)**	2	-
**Urethral plaque**	14	4
**Bladder stones**	3	1
**Urethritis**	2	-
**Acute Renal Failure**	8	3
**Chronic Renal Failure**	6	2
**Diabetes Mellitus**	2	1
**Other diseases**	9	2

### Complete Blood Count (CBC) and blood serum biochemistry

All the animals in the study were found to be within the normal range of CBC parameters. At the same time, leucocytosis was not observed in 16 culture-positive cats. Biochemical blood serum values (Serum glucose, blood urea nitrogen, creatinine, alanine aminotransferase, aspartate aminotransferase) were determined for every patient. In our study, these parameters were found not to be significant.

### Medical imaging

Blood clot formation was seen in the urinary bladder in cats diagnosed with haemorrhagic cystitis. The echogenicity of the clots was variable (hypo- to hyper-) and the bladder wall was thickened. Bladder stones were seen hyperechoic and distal shadows were detected. The bladder wall was also thickened in cats diagnosed with renal failure and cystitis. No abnormal findings were seen in cats with urethritis. The sonographic appearance of transitional cell carcinoma was irregular, its shape was irregular and the echogenicity seemed non-homogenous. The ultrasonographic findings are compatible with our diagnosis.

### Urine analysis microscopic evaluation

Leucocyte numbers detected by urine microscopic evaluation are summarized in Table 2.

**Table 2:** **Number of leucocytes detected by urine microscopic examination**

**Leucocytes/HPF**	**Number of cats**	**culture-positive cats**
**5-6**	11	2
**7-10**	6	1
**10-15**	23	5
**≥15**	21	8

**Figure 1:**
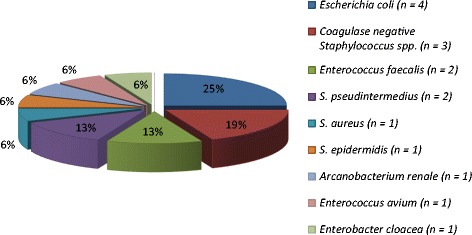
**Type and number of bacterial species isolated in cats.**

Bacterial growth was observed in 16 of 61 (26.2%) cats urine samples with a leucocyte count ≥ 5–6 leucocytes (Figure 1). All the isolates were pure cultures.

### Bacterial growth and susceptibility testing

Bacterial growth was observed in 5 of 20 (25%) urine samples of female cats and in 11 of 41 (26.8%) samples of male cats (*p* > 0.05). Three in five cats (60%) with bacterial LUTI were 1 year old or younger, 9/38 (23.6%) 2–7 years old and 4/18 (22.2%) 8 years old or older (*p* > 0.05). In this study, no significant difference was found between female and male cats with bacterial LUTI (*p* = 0.879). The differences among age groups were also not significant (*p* = 0.200).

Antimicrobial susceptibility tests results showed that 13 of 16 (81%) isolates were susceptible to cefovecin. *E. avium* and *S. epidermidis* isolates were resistant and *Arcanobacterium renale* isolate was intermediate.

## Discussion and Conclusions

Incorrect therapy of urinary tract disease, overuse and misuse of antimicrobials can have negative effects on patient health (e.g. failure to resolve infections), the allocation of resources (e.g. need for repeated or prolonged treatment), and public health (e.g. antimicrobial resistance) and may raise regulatory concerns (e.g. antimicrobial use) [13]. The antimicrobial activity of cefovecin is similar to that of other cephalosporin antibiotics, which share low toxicity and good activity against many Gram-positive and Gram-negative aerobic bacteria [6].

Bacterial urinary tract infections (UTIs) in cats are relatively rare [14]. Studies of cats with clinical signs of lower urinary tract disease (dysuria, stranguria, pollakiuria) have consistently shown that the overall prevalence of positive bacterial urine cultures is <3% [2,15]. Some studies have reported much higher prevalence rates (15–43%) in cats that have their urinary tract defence mechanisms compromised by the effects of other diseases and/or by the treatment [2]. In this study, bacterial growth was observed in 26.2% of cats’ urine samples with ≥ 5–6 leucocytes. Our findings confirm to a large extent to the results reported by Weese et al. [15].

Bacteriuria is generally seen in older cats [16]. In our study, the percentage of young cats (≤1 years old) was highest. Most of the younger cats in our sample group spent time both indoor and outdoor. They generally hunted and usually drank water from flower bowls and ponds. These risk factors and their close contact with stray cats, may have contributed to the high prevalence of urinary problems in this age group compared to indoor and older cats.

Urine analyses were shown to be a useful indicator for UTIs. The most commonly isolated bacteria of cats with urinary tract infections were reported to be *Escherichia coli, Enterococcus* spp., *Staphylococcus* spp. and *Streptococcus* spp. [4,6,9]. Our results support these findings.

Proper and timely diagnosis is critical for the treatment of lower urinary tract infections as well as for the selection of appropriate antimicrobials and drugs. Cefovecin has been specifically developed for the animal practice as a long-acting third-generation cephalosporin with duration of action of 14 days. Stegemann et al. [4] reported that cefovecin exhibited a broad activity against a range of Gram-negative pathogens and was not active in vitro against *P. aeruginosa*. Wernick and Müntener [5] have reported that cefovecin showed no bactericidal activity against *Enterococcus* spp. but it is active against *Arcanobacterium renale.* Our results (81% of isolates susceptible to cefovecin) are in agreement with these findings. However, *E. avium* isolate was found to be resistant, and *Arcanobacterium renale* isolate to be of intermediate susceptible to cefovecin. Stegemann et al. [4] reported that cefovecin was not appreciably active against *Enterococcus* spp., although in this study *E. faecalis* isolates were found to be susceptible in vitro. While literature suggests good activity of cefovecin against coagulase-negative staphylococci, in this study *S. epidermidis* isolate was resistant to cefovecin [4,8].

It is known that antimicrobials are the cornerstone of LUTI therapy. Despite the high cost of cefovecin in Turkey, its effectiveness and usefulness have been discussed in this study. Cefovecin is one of the antimicrobial agents that can be used in lower urinary tract infections and it is easy to administer a single injection.
